# Supplementary material for: PPARG is central to the initiation and propagation of human angiomyolipoma, suggesting its potential as a therapeutic target
Source: EMBO Mol Med. 2017 Mar 8;9(4):508–30. doi: 10.15252/emmm.201506111 (PMC5376758; doi:10.15252/emmm.201506111)
Supplement: Supplementary file 1 — Appendix [file EMMM-9-508-s001.pdf]

PPARG IS CENTRAL TO THE INITIATION AND PROPAGATION OF HUMAN ANGIOMYOLIPOMA, SUGGESTING ITS POTENTIAL AS A THERAPEUTIC TARGET

Table of Contents

Supplementary methods 2
Supplementary figures 5
Appendix Figure S1 5
Appendix Figure S2 6
Appendix Figure S3 7
Appendix Figure S4 8
Appendix Figure S5 9
Appendix Figure S6 11
Appendix Figure S7 11
Appendix Figure S8 12
Appendix Figure S9 14
Appendix Figure S10 15
Appendix Figure S11 16
Supplementary tables 17
Appendix Table S1 17
Appendix Table S2 18
Appendix Table S3 20
Appendix Table S4 21
Appendix Table S5 22
Appendix Table S6 24
Appendix Table S7 25
Appendix Table S8 26

Supplementary methods

Hematoxylin and eosin (H&E) staining

5 μm sections of paraffin-embedded Xn tissues were mounted on super frost/plus glass and incubated at 60°`C for 40 minutes. After de-paraffinization, slides were incubated in Mayer's Hematoxylin solution (Sigma-Aldrich) and incubated with 1% Hydrochloric acid in 70% ethanol for 1 minute. Slides were then incubated for 10 seconds in Eosin (Sigma-Aldrich). Images were produced using Olympus BX51TF fluorescent microscope with Olympus DP72 camera and cellSens standard software.

Immunohistochemical (IHC) and immunofluorescent (IF) staining of paraffin-embedded Xn tissues

Sections were pre-treated using OmniPrep solution (Zytomed Systems) in 95°C for 1 hour according to the manufacturer's protocol. For IHC, blocking was performed using Cas-Block solution (Invitrogen) for 20 minutes followed by 1 hour of incubation at room temperature (RT) with primary antibodies for HLA, α-SMA, CD31, HMB-45, PPARγ and pS6 **(Appendix Table S6)**. Samples were incubated with secondary antibodies (ImmPRESS^{TM} anti mouse/rabbit reagent peroxidase, Vector labs) for 30 minutes at RT and detected using ImmPACT DAB kit (Vector) according to the manufacturer's protocol. Hematoxylin was used for counterstaining. For IF, sections were blocked with Cas-Block solution (Invitrogen) for 1 hour followed by incubation with primary antibodies for HLA and CD31 **(Appendix Table S6)** and then washed and incubated with secondary antibodies **(Appendix Table S7)** for 1 hour. Mounting containing DAPI (Southern Biotech) was applied. Photos were obtained using Olympus BX51TF fluorescent microscope with Olympus DP72 camera and cellSens standard software.

IF staining of cells

Cells were grown on coverslips in 24-well culture dishes overnight, after which they were washed with PBS, fixed in 3% paraformaldehyde (PFA) for 10 minutes at RT and washed with PBS 3 times for 5 minutes at RT. The cells were next permeabilized for 5 minutes with 0.2% (vol/vol) Triton X-100 in PBS at RT, washed 3 times with PBST (PBS with 0.05% Tween-20)

for 5 minutes and then blocked with Cas-Block solution (Invitrogen) for 1 hour at RT. Next, the cells were incubated with the primary antibody (PPARg, Santa-Cruz, and β-Actin, Cell signaling) for 1 hour at RT, washed 3 times with PBST, incubated with the secondary antibody (as described above) for 1 hour at RT and washed three times with PBST. Finally, mounting with DAPI (Southern Biotech) was applied. Photos were obtained using Olympus BX51TF fluorescent microscope with Olympus DP72 camera and cellSens standard software.

Flow Cytometry

10^5 Cells were suspended in FACS buffer (0.5% BSA, 2 mM EDTA in 1× PBS) and blocked with FcR blocking reagent (MiltenyiBiotec) and human serum (1:1). The cells were then incubated with a primary antibody or an isotype control **(Appendix Table S8)**. Cells were incubated with a secondary antibody if needed **(Appendix Table S8)**. Cell viability was tested using 7AAD viability staining solution (eBioscience). Cell labelling was detected using FACSCalibur (BD Pharmingen). FACS results were analyzed using FlowJo analysis software. For flow cytometry analysis of AML Xn, freshly removed Xn were dissociated into a single cell suspension as described above and immediately analyzed.

Quantitative real-time RT-PCR analysis of gene expression

Total RNA was isolated using TRIzol reagent (Life Technologies) according to the manufacturer's instructions. cDNA was synthesized using the High Capacity cDNA reverse transcription kit (Applied Biosystems, ABI). Quantitative Real-time RT-PCR (qRT-PCR) was performed using StepOnePlus Real-Time PCR System (ABI) and the specific TaqMan Gene Expression assays for the relevant genes in the presence of TaqMan Fast Universal PCR Master Mix (both from ABI). hHPRT1, hGAPDH and hB2M were used as endogenous controls. The results were analyzed using StepOnePlus Real-time Software in the ΔΔCT method which determines fold change in gene expression relative to a comparator sample. Statistical analysis was performed using a non-paired 2-tailed t-test. Statistical significance was considered to be p-value <0.05.

Human FK and AK tissue

Normal human 16 week gestation kidney was obtained following curettage of elective abortions. Normal human AK samples were retrieved from borders of renal cell carcinoma tumors from patients who underwent partial nephrectomy. Fetal and adult kidney tissues were handled within 1 h following the curettage or nephrectomy procedures, respectively. Collected tissues were washed with cold Hank's Balanced Salt Solution (HBSS) (Invitrogen, Carlsbad, Calif., USA) and cut into 0.5 cm cubes by sterile surgical scalpels. The pieces were then used for total RNA extraction with TRIzol (Life Technologies, Invitrogen, Carlsbad, Calif., USA).

Cell Culture

AML cell lines and Xn cells were grown in DMEM (Invitrogen) supplemented with 10% fetal bovine serum (Invitrogen), 1% Penicillin–streptomycin 100M and 1% L-glutamine (both from Biological Industries). Human AK and FK and WT cells were grown as previously described (Buzhor et al, 2013; Harari-Steinberg et al, 2013; Pode-Shakked et al, 2013). Human bone marrow-derived MSCs were isolated and grown as previously described (Resnick et al, 2013). The cells demonstrated the typical expression of surface markers and differentiated into adipocytes and osteoblasts under appropriate culture conditions (**Appendix Figure S11**).

Supplementary figures

Appendix Figure S1

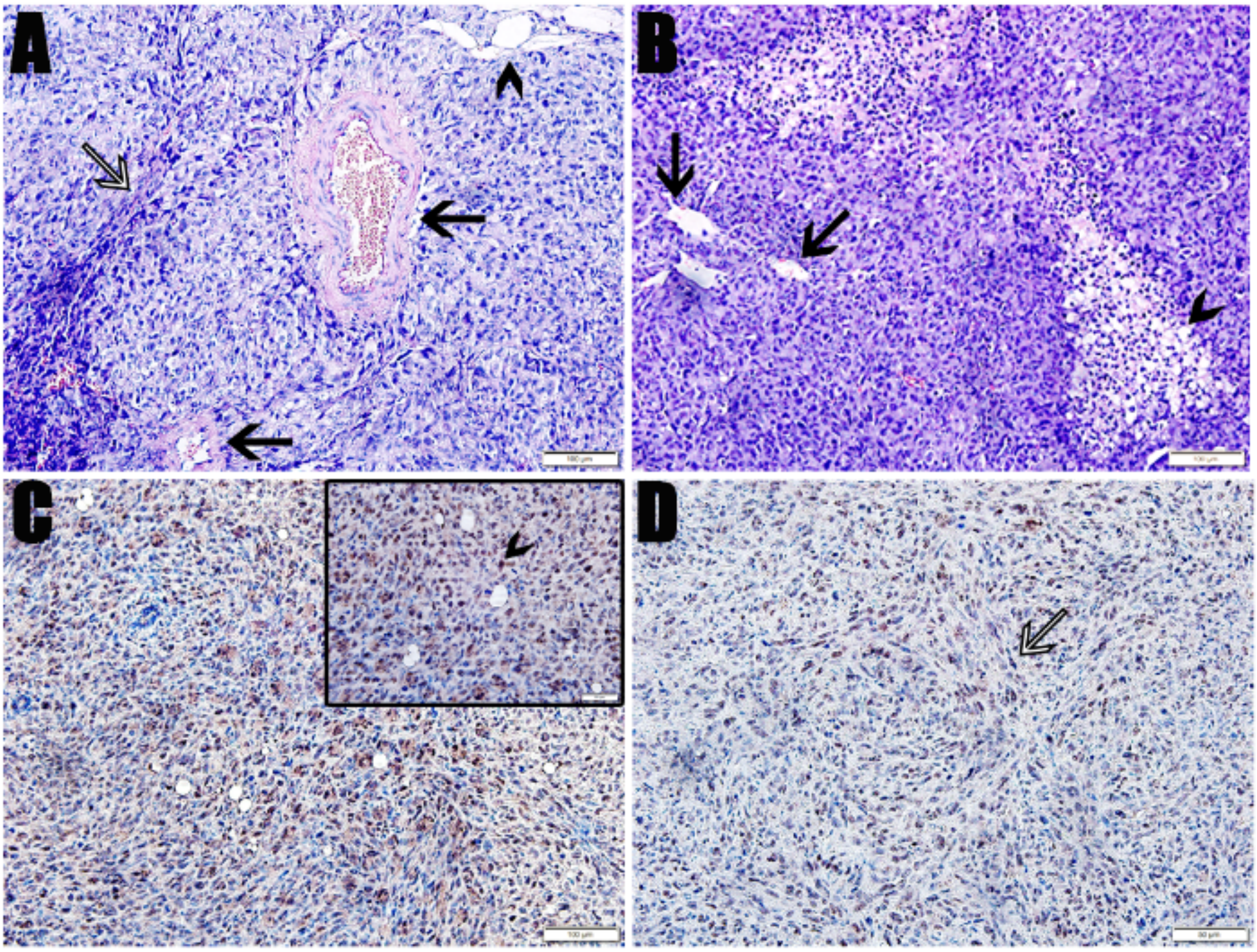

Appendix Figure S1: Validation of the human AML-Xn model: Shown are two representative repetitions of the newly established Xn model, entitled UMB2-Xn and UMB3-Xn. Shown is the histology of the 5th (T5) generation of Xn in mice. Both UMB2-T5 **(A)** and UMB3-T5 **(B)** demonstrate in H&E staining, the same histological features consistent with the parental tumor, namely the presence of blood vessels (black arrows), adipocytes (arrowheads) and spindle-shaped, early myoid cells (white arrow). In addition, these tumors as well demonstrate strong nuclear expression of PPARG **(C&D)**. Notably, PPARG expression PPARG is robustly expressed in the mass of undifferentiated cells of the tumors **(C, insert)**. In addition, PPARG is expressed in both adipocytic and myoid cells within the tumors **(D, arrow)**. Scale bar: 100 µm (except for insert, 50 µm).

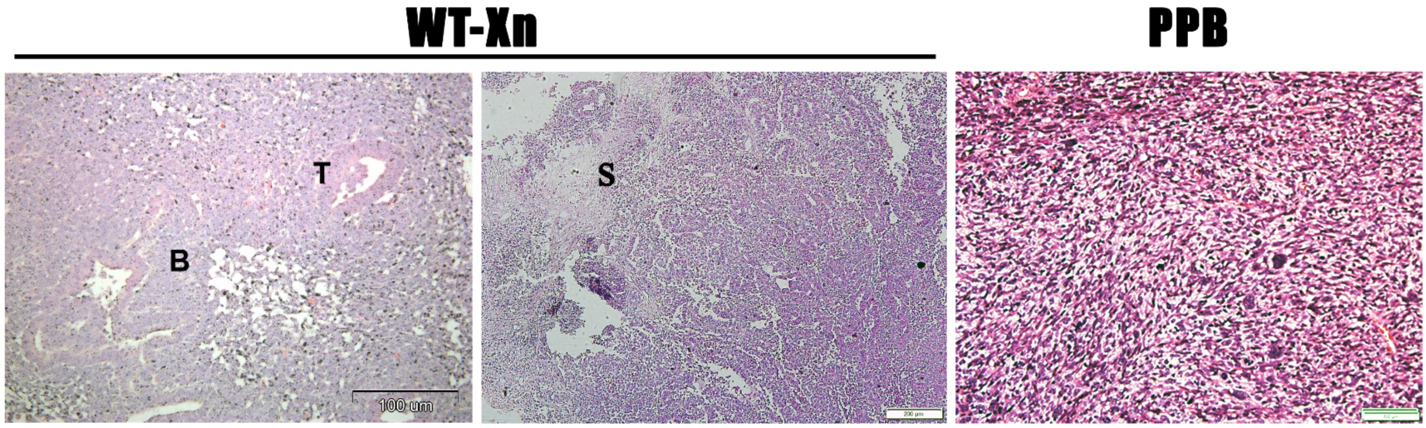

Appendix Figure S2: H&E staining of Wilms' tumor (WT) and Pleuro-Pulmonary Blastoma: In order to demonstrate the specificity of the human AML-Xn model, we have characterized Xn tumors of two different tumors: WT and PPB, representing a renal and non-renal tumors. Notably, both Xn models exhibit a significantly different histological appearance compared to the AML-Xn model, which is at the same time reminiscent of the parental neoplasm. WT-Xn (left and middle panels) demonstrate the classical tri-phasic histology, consisting of renal tubules (T), dense blastema (B) and stroma (S). In contrast, PPB-Xn (right panel) exhibit the typical hyper-cellular primitive blastemal-like morphology.

Appendix Figure S3

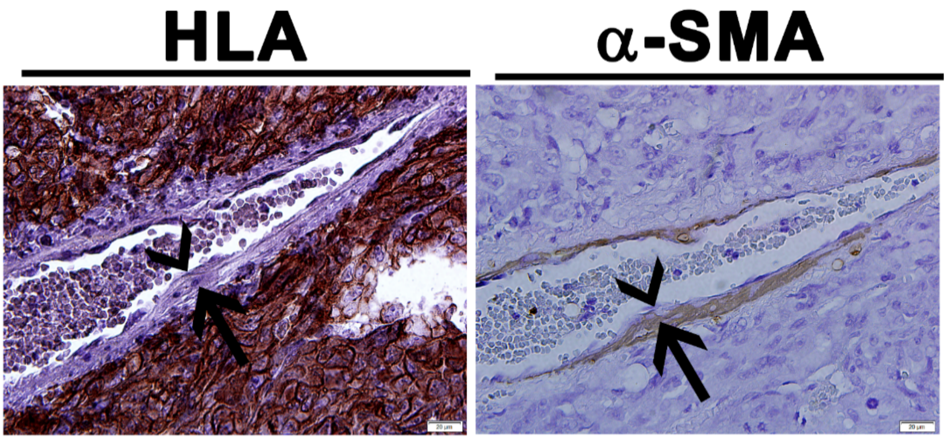

Appendix Figure S3: Immunohistochemical staining of T4 (4th generation)-xenografts (Xn) for HLA **(Left panel)** and α-SMA **(Right panel)**, demonstrating mouse-derived blood vessels within the human-derived tumor, in which both the endothelial lining **(arrowheads)** and the α-SMA^{+} perivascular cells **(arrows)** are HLA^{-}. In contrast, the Xn also harbors vessels in which perivascular cells are of human origin, thus expressing both HLA and α-SMA **(see Figure 1D)**. Shown are sequential sections. Scale bar: 20 μm

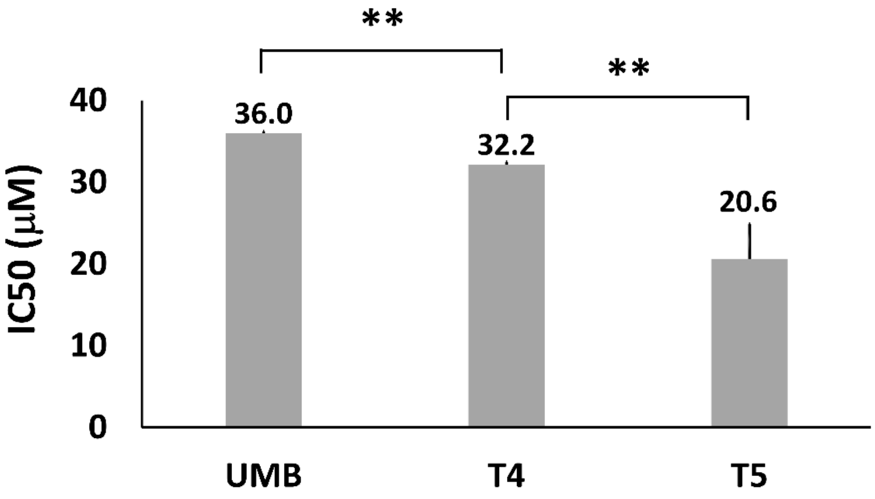

Appendix Figure S4: Inhibitory concentration 50 (IC50) of the PPARG inhibitor GW9662 in the AML cell line UMB and subsequent 4th and 5t generations of Xn tumors (T4 and T5, respectively). Results shown as mean±SD (n=3).*, $p<0.05$; **, $p<0.01$ (2-tailed Student's t test).

Appendix Figure S5

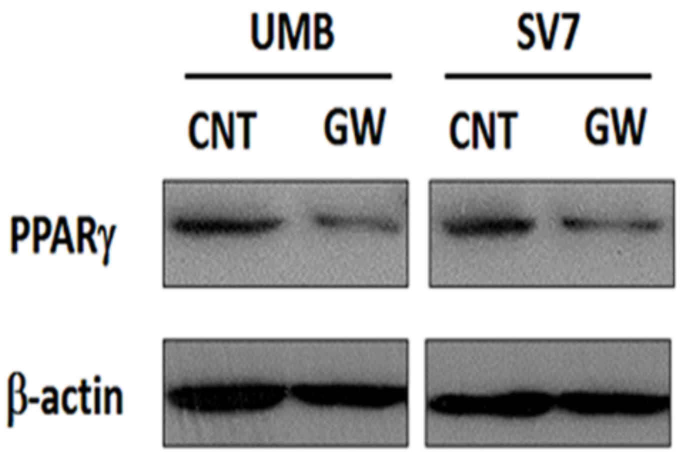

Appendix Figure S5: Western blot analysis demonstrating PPARγ expression in UMB and SV7 cells treated with GW9662 (GW) compared to control cells (CNT).

Appendix Figure S6

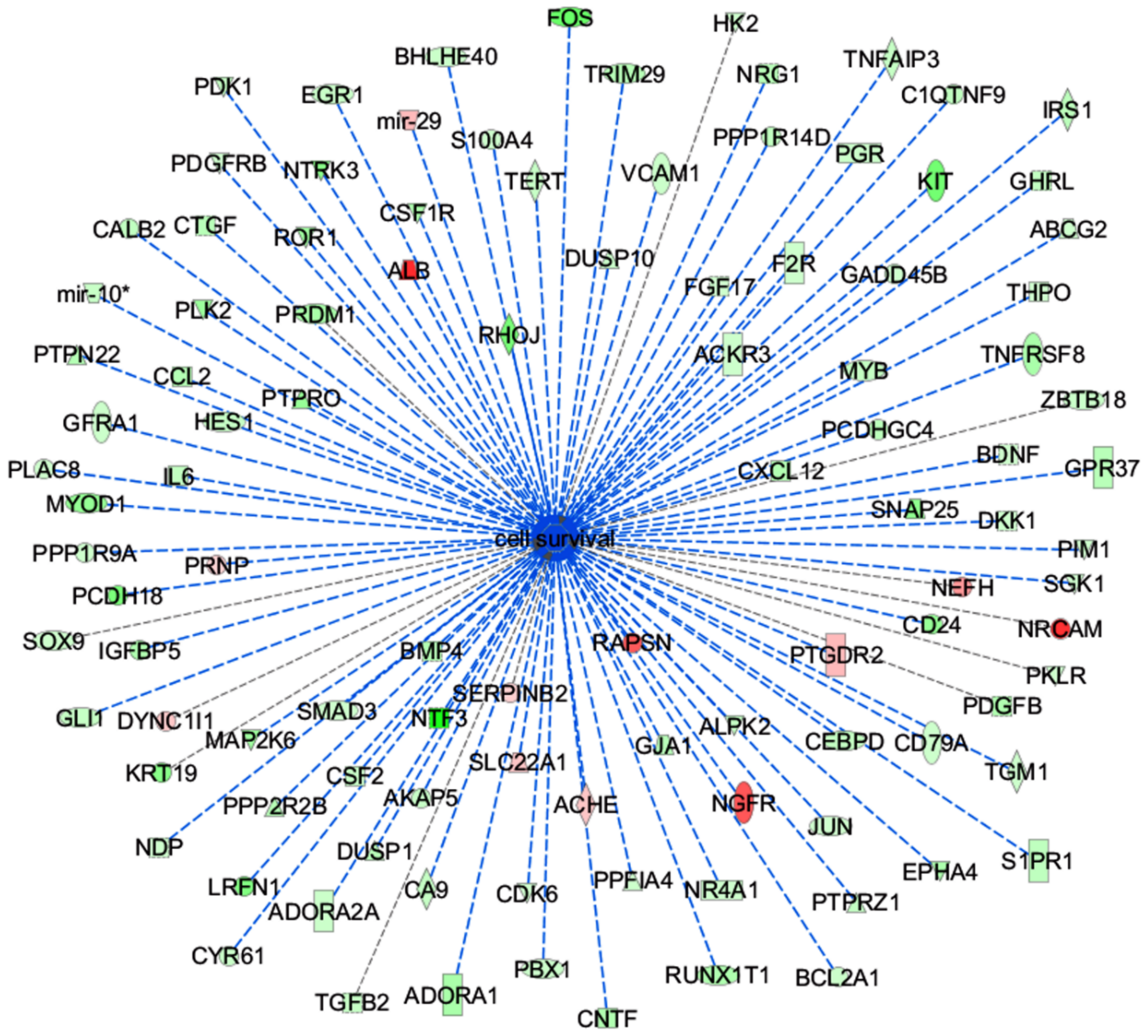

<u>Appendix Figure S6:</u> Ingenuity pathway analysis (IPA) of differentially-expressed genes leading to the decreased cell survival seen in AML cells treated for 24h with GW9662 compared with control, vehicle-treated cells. Genes in green were down-regulated while genes in red were up-regulated, with darker colors indicating a more significant change.

Appendix Figure S7

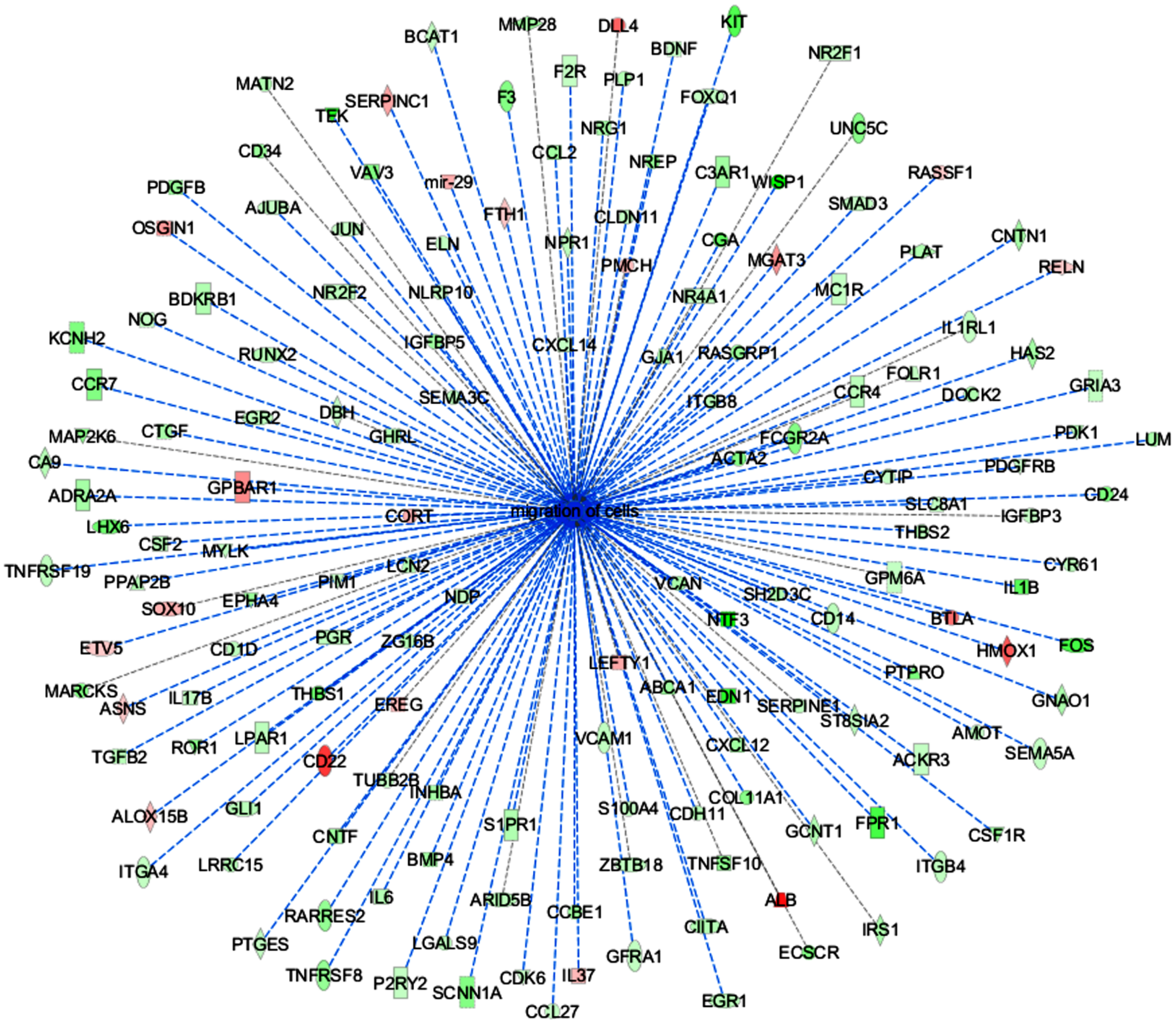

Appendix Figure S7: Ingenuity pathway analysis (IPA) of differentially-expressed genes leading to the decreased migration of cells seen in AML cells treated for 24h with GW9662 compared with control, vehicle-treated cells. Genes in green were down-regulated while genes in red were up-regulated, with darker colors indicating a more significant change.

Appendix Figure S8

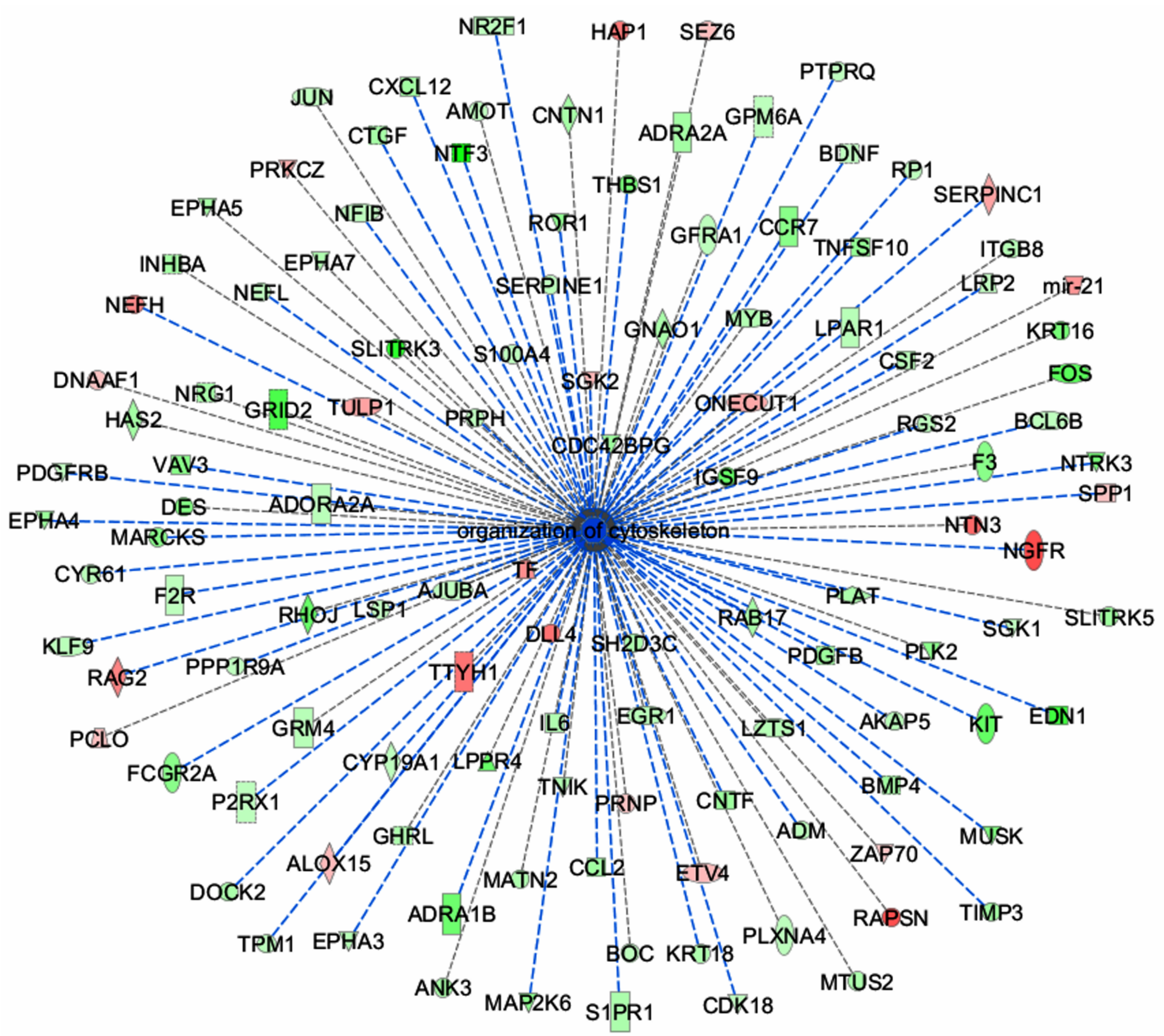

Appendix Figure S8: Ingenuity pathway analysis (IPA) of differentially-expressed genes leading to the decreased organization of cytoskeleton seen in AML cells treated for 24h with GW9662 compared with control, vehicle-treated cells. Genes in green were down-regulated while genes in red were up-regulated, with darker colors indicating a more significant change.

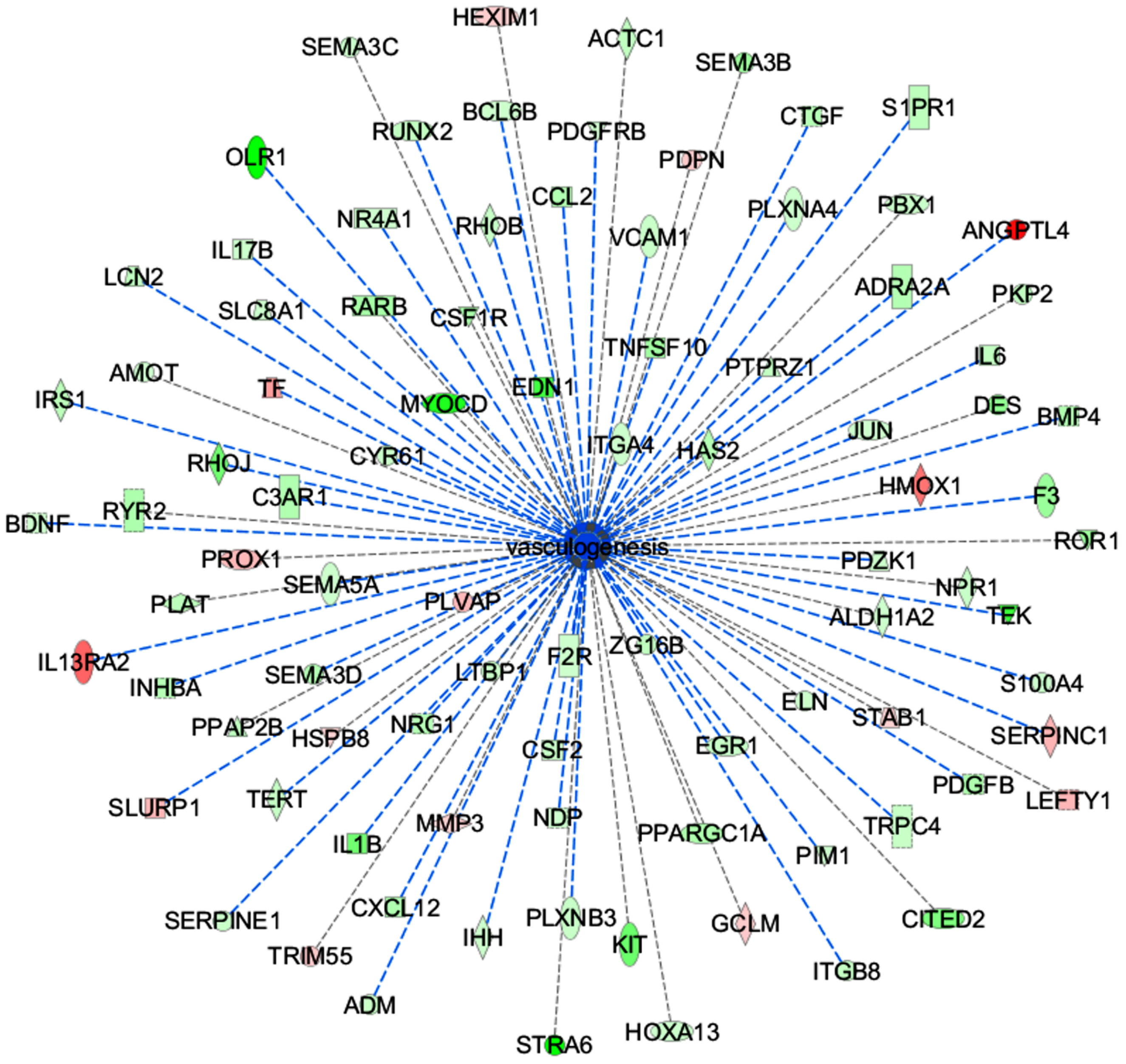

Appendix Figure S9: Ingenuity pathway analysis (IPA) of differentially-expressed genes leading to the decreased vasculogenesis cell survival seen in AML cells treated for 24h with GW9662 compared with control, vehicle-treated cells. Genes in green were down-regulated while genes in red were up-regulated, with darker colors indicating a more significant change.

Appendix Figure S10

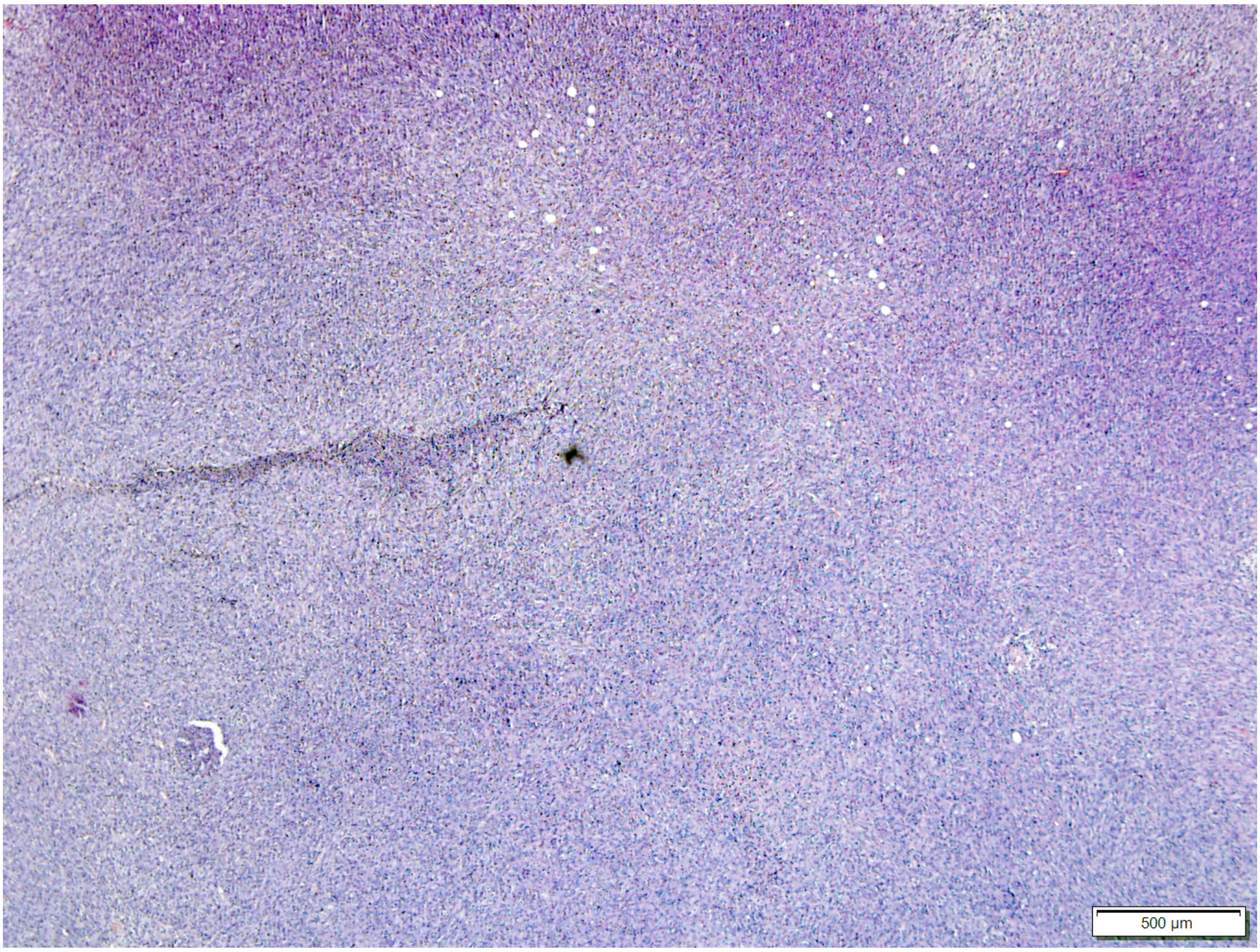

Appendix Figure S10: Representative low magnification photograph of T5-Xn, demonstrating the general composition of the Xn. Most of the tumor is seen to consist of dense masses of un-differentiated small hyperchromatic cells, alongside blood vessels, adipocytes and myoid areas.

Appendix Figure S11

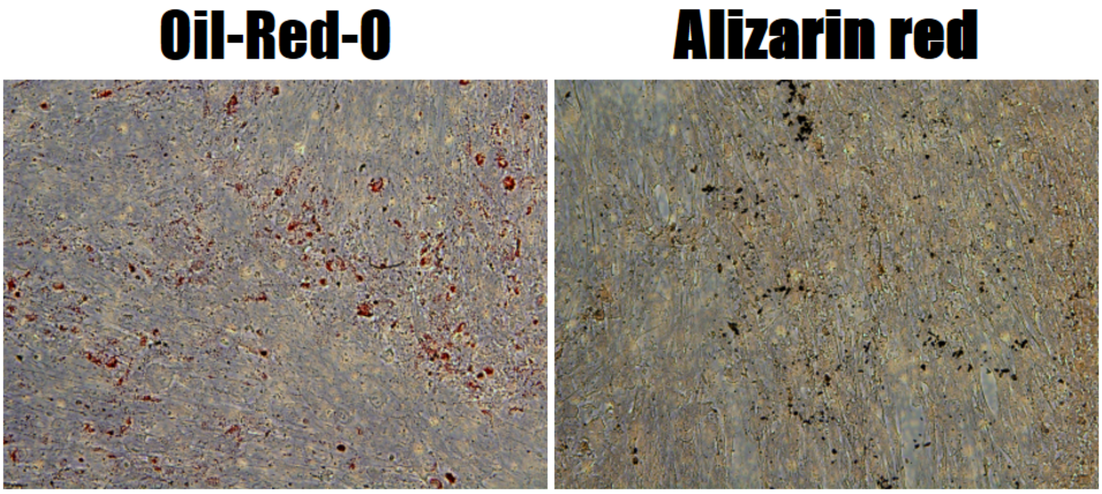

Appendix Figure S11: Multipotent mesenchymal stromal cells (MSCs) demonstrating multipotency towards the adipogenic and osteogenic lineages, as manifested by the positive Oil-red-O (left) and Alizarin red (right) stainings. Scale bar: 500 μm.

Supplementary tables

Appendix Table S1

Gene symbol	Function	Fold change
FABP4	AML phenotype	152.52
FABP5	AML phenotype	12.17
FGF2	AML phenotype	3.4
PLIN2	AML phenotype	3.12
SERPINH1	AML phenotype	3.11
SERPINE1	AML phenotype	2.08
PPARGC1A	AML phenotype	2.04
VEGFA	AML phenotype	1.94
SERPINF1	AML phenotype	-1.91
TJP1	AML phenotype	-2.24
WNT5A	AML phenotype	-5.4
ABCG2	Tumor growth	3.65
CCND1	Tumor growth	3.29
BIRC5	Tumor growth	2.63
FOSL1	Tumor growth	2.58
CDC42	Tumor growth	2.42
CDK5	Tumor growth	2.35
KRAS	Tumor growth	2.05
TP53	Tumor growth	-1.89
CASP3	Tumor growth	-2.01
EP300	Tumor growth	-3.71
TNFSF10	Tumor growth	-3.73
CDKN1C	Tumor growth	-4.2

FKBP4	mTOR pathway	7.2
PTEN	mTOR pathway	2.42
PRKAA2	mTOR pathway	1.95
AKT2	mTOR pathway	-2.08
PDPK1	mTOR pathway	-2.29
INSR	mTOR pathway	-2.37
IRS2	mTOR pathway	-3.16
AKT1	mTOR pathway	-3.54
IRS1	mTOR pathway	-4.37
CEBPB	Transcriptional regulation	2.39
CEBPD	Transcriptional regulation	1.97
MED6	Transcriptional regulation	1.81
CREB1	Transcriptional regulation	-1.72
PLAGL1	Transcriptional regulation	-2.05
STAT3	Transcriptional regulation	-2.11
TRRAP	Transcriptional regulation	-2.15
TBP	Transcriptional regulation	-2.19
MECP2	Transcriptional regulation	-2.3
EGR1	Transcriptional regulation	-3.66

Appendix Table S1: Ingenuity pathway analysis (IPA) of PPARG-dependent genes demonstrating significant change between T1-Xn and T5-Xn according to cellular functions.

Appendix Table S2

Gender	Age	Histology	HMB-45	αSMA	Features
F	27	Triphasic	NA	NA	TSC-related
F	19	Triphasic	+	+	
F	66	Epithelioid	+	+	Atypical, aggressive
F	69	Blood vessel predominant, fat and muscle poor	+	NA	
F	36	Muscle predominant, fat poor	+	+	
M	64	Fat predominant	+	+	Retroperitoneal AML
F	75	Triphasic	+	NA	

Appendix Table S2: Summary of primary AML specimens used in this work. F, female; M, male; NA, not available; TSC, Tuberous Sclerosis Complex.

GO term	FDR	Count
Cell motion	6.47E-05	78
Regulation of blood vessel size	3.85E-04	19
Regulation of angiogenesis	1.45E-03	20
Cell projection morphogenesis	1.55E-03	46
Regulation of cell proliferation	2.70E-03	103
Cell morphogenesis	2.70E-03	59
Cell-cell adhesion	1.81E-02	47
Cell fate commitment	3.14E-02	29

Appendix Table S3: Enriched GO (Gene ontology) functions in AML cells treated for 24h with GW9662.

Appendix Table S4

Upstream regulator	Z-score	p-Value
TGFB1	-4.026	7.30E-37
NFkB	-3.535	3.36E-11
EDN1	-3.431	5.87E-08
Ap1	-3.094	3.53E-05
PDGF BB	-3.025	6.83E-18
Vegf	-3.004	4.57E-11
CTNNB1	-2.951	5.89E-20
LEP	-2.711	2.02E-06
PTK2	-2.383	3.78E-05
TGFBR1	-2.381	5.48E-05
TGFB3	-2.291	5.70E-12
LEPR	-2.219	4.23E-04
Pdgf complex	-2.17	6.04E-09
PGF	-2.158	3.46E-06
ERK1/2	-2.13	3.46E-11
SMAD3	-2.086	2.83E-21
Let-7	2.168	4.48E-02
KLF2	2.178	6.54E-07
SOCS1	2.236	3.45E-02

Appendix Table S4: Ingenuity pathway analysis (IPA) demonstrating significantly changed upstream regulators in in AML cells treated with GW9662 compared with control, vehicle-treated cells. Positive Z-scores represent activated upstream regulators, while negative Z-scores represent inhibited upstream regulators.

Appendix Table S5

Figure	p-value
1A	p(T2)= 2.66E-07;<br>P(T3)=0.046<br>p(T4)=0.077
4A	p(UMB,86.5)=0.0001<br>p(UMB, 27.0)=8.84E-13<br>p(UMB,13.1)=1.32E-08<br>p(SV7, 83.9)= 0.001<br>p(SV7,83.3)=2.82E-06<br>p(T4,82.9)= 0.007<br>p(T4,68.1)=0.003
4D	p=0.00007
4G	p(SV7)=5.59E-05;<br>p(UMB)=0.00023<br>p(T4)= 0.007;<br>p(AK)=0.003
4H	p(266.7)= 2.91E-06;<br>p(297.2)= 0.036<br>p(331.0)=0.047
5B	p(48h, 2.6)=0;<br>p(96h, 2.1)=0.001<br>p(48h, 2.1)=0.013;<br>p(96h, 2.4)=0.007
6A	p(7)= 0.0385;<br>p(11)=0.00004<br>p(15)=0.0001
6D	p=0.018
6E	p=0.005
6F	p=0.004
7D	p(UMB,0.55)= 0.0009;<br>p(SV7,0.23)=0.0006<br>p(T5,0.33)=0.0196;<br>p(UMB,0.37)=0.00004<br>p(SV7, 0.52)=0.003;<br>p(T5,0.11)=0.0003
8C	p(SIX2,10.6)=0.033;<br>p(OSR1,21.7)=0.049<br>p(PAX2,4.8)=0.0025;<br>p(CITED1,20.2)=0.0134<br>p(FOXD1, 3.6)=0.0113;<br>p(SIX2,0.1)=0.0001<br>p(PAX2, UMB)=0.0001;<br>p(PAX2, SV7)=0.0001<br>p(CITED1, 0.2)=0.0091;<br>p(FOXD1,UMB)=0.0001<br>p(FOXD1,SV7)=0.0006;<br>p(PAX2,0.1)=0.0001
8D	p=0.0295
8E	p(CD73)= 0.0039;<br>p(CD90)=0.011<br>p(CD105)=0.0003
S4	p(T4 vs. UMB)= 0.0004;<br>p(T5 vs. T4)= 0.0098
S6	p(75.0)=0.00096;<br>p(85.5)=2.6E-05<br>p(53.9)=3.31E-07;<br>p(52.5)=1.69E-08<br>p(82.1)=0.0016;<br>p(79.8)=0.0003
S7	p=0.0499
S8	p=1.16E-07

Appendix Table S5: p-values obtained in all statistical analyses carried out in this work.

Appendix Table S6

Target	Manufacturer	Catalog number	Dilution
HLA	Abcam	ab52922	1:200
α-SMA	Dako	IR611	1:100
CD31	Dako	M0823	1:100
HMB-45	Dako	M0634	1:100
PPARG	Santa Cruz Biotechnology	sc-7196	IHC/IF:1:50<br>WB:1:200
Phospho-S6	Cell Signaling Technology	2211	1:400
β-actin	Cell Signaling Technology	4970s	IF:1:200<br>WB:1:1,000

Appendix Table S6: Primary antibodies used in immunohistochemical and immunofluorescent stainings and western blot (IHC, IF and WB, respectively).

Appendix Table S7

Antibody	Manufacturer	Catalog number	Dilution
Alexa Fluor 488 donkey anti-rabbit	Invitrogen	A-11008	1:1,200
Alexa Fluor 550 donkey anti-mouse	Invitrogen	A-31570	1:1,200

Appendix Table S7: Secondary antibodies used in immunohistochemical and immunofluorescent stainings.

Antibody	Manufacturer	Catalog number
Mouse anti human CD14 (FITC)	BD biosciences	555397
Mouse anti human CD73 (PE)	BD biosciences	550257
Mouse anti human CD90 (PE)	BD biosciences	562385
Mouse anti human CD31 (FITC)	eBioscience	11-0319-41
Mouse anti human CD45 (APC)	eBioscience	17-9459-41
Mouse anti human CD105 (APC)	eBioscience	17-1057-41
Mouse anti human NCAM1 (PE)	eBioscience	12-0567-41
Mouse anti human CD34 (FITC)	IQ Products	IQP-144F50

Appendix Table S8: Antibodies used for flow cytometry experiments.
