# Supplementary material for: PPARG is central to the initiation and propagation of human angiomyolipoma, suggesting its potential as a therapeutic target
Source: EMBO Mol Med. 2017 Mar 8;9(4):508–30. doi: 10.15252/emmm.201506111 (PMC5376758; doi:10.15252/emmm.201506111)
Supplement: Supplementary file 2 — Expanded View Figures PDF [file EMMM-9-508-s002.pdf]

Expanded View Figures

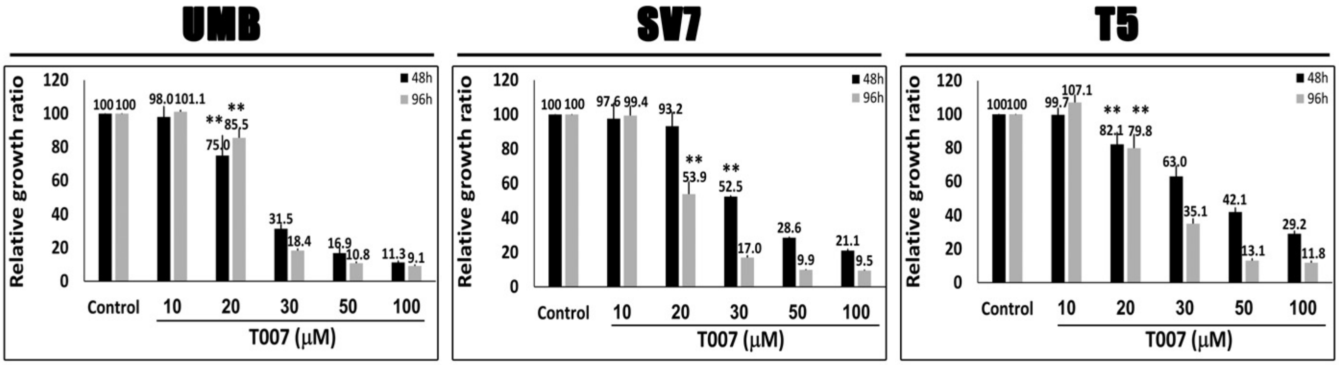

Figure EV1. The effect of the PPARG antagonist T007 on the growth of UMB, SV7 and T5-Xn cells.
Relative growth ratios of UMB, SV7, and T5 (5th generation)-xenograft (Xn) cells (left, middle, and right panels, respectively) treated with the PPARG inhibitor T007 in varying concentrations, compared to control, vehicle-treated cells. Results shown as mean ± SD (n = 3). $^{**}P$ < 0.01 (one-way ANOVA with Bonferroni *post hoc* test). The exact *P*-values are specified in Appendix Table S5.

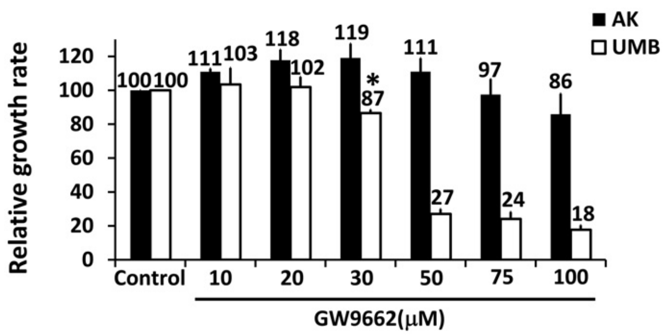

Figure EV2. The effect of the PPARG antagonist GW9662 on the growth of UMB and AK cells.
Relative growth ratios of primary human adult kidney cells (AK) and UMB cells treated with varying concentrations of GW9662 for 48 h. Vehicle-treated cells served as control. Results shown as mean ± SD (n = 3). $^{*}P$ < 0.05 (one-way ANOVA with Bonferroni *post hoc* test). The exact *P*-values are specified in Appendix Table S5.

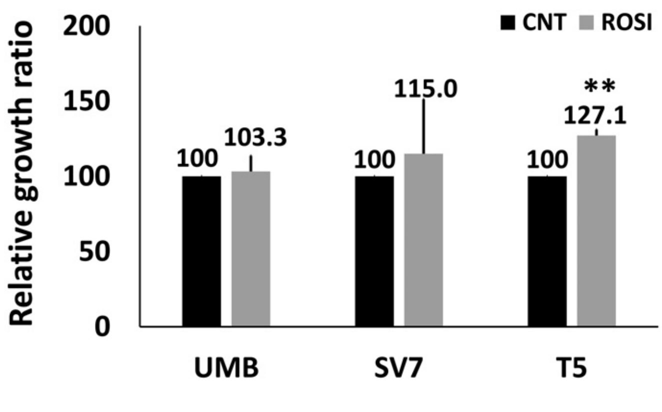

Figure EV3. The effect of the PPARG agonist rosiglitazone on the growth of UMB, SV7, and T5-Xn cells.
The three cell types were treated with either rosiglitazone (ROSI) or vehicle (CNT) and assessed for relative growth ratio following 48 h. Results shown as mean ± SD (n = 3). $^{**}P$ < 0.01 (one-way ANOVA with Bonferroni *post hoc* test). The exact *P*-values are specified in Appendix Table S5.

© 2017 The Authors
